# Genomic analysis of *P* elements in natural populations of *Drosophila melanogaster*

**DOI:** 10.7717/peerj.3824

**Published:** 2017-09-15

**Authors:** Casey M. Bergman, Shunhua Han, Michael G. Nelson, Vladyslav Bondarenko, Iryna Kozeretska

**Affiliations:** 1 Faculty of Life Sciences, University of Manchester, Manchester, United Kingdom; 2 Department of Genetics and Institute of Bioinformatics, University of Georgia, Athens, GA, United States of America; 3 Institute of Bioinformatics, University of Georgia, Athens, GA, United States of America; 4 Department of General and Molecular Genetics, Taras Shevchenko University of Kyiv, Kyiv, Ukraine

**Keywords:** *P* element, *Drosophila melanogaster*, Transposable elements, Population genomics, Hybrid dysgenesis

## Abstract

The *Drosophila melanogaster P* transposable element provides one of the best cases of horizontal transfer of a mobile DNA sequence in eukaryotes. Invasion of natural populations by the *P* element has led to a syndrome of phenotypes known as P-M hybrid dysgenesis that emerges when strains differing in their *P* element composition mate and produce offspring. Despite extensive research on many aspects of *P* element biology, many questions remain about the genomic basis of variation in P-M dysgenesis phenotypes across populations. Here we compare estimates of genomic *P* element content with gonadal dysgenesis phenotypes for isofemale strains obtained from three worldwide populations of *D. melanogaster* to illuminate the molecular basis of natural variation in cytotype status. We show that *P* element abundance estimated from genome sequences of isofemale strains is highly correlated across different bioinformatics approaches, but that abundance estimates are sensitive to method and filtering strategies as well as incomplete inbreeding of isofemale strains. We find that *P* element content varies significantly across populations, with strains from a North American population having fewer *P* elements but a higher proportion of full-length elements than strains from populations sampled in Europe or Africa. Despite these geographic differences in *P* element abundance and structure, neither the number of *P* elements nor the ratio of full-length to internally-truncated copies is strongly correlated with the degree of gonadal dysgenesis exhibited by an isofemale strain. Thus, variation in *P* element abundance and structure across different populations does not necessarily lead to corresponding geographic differences in gonadal dysgenesis phenotypes. Finally, we confirm that population differences in the abundance and structure of *P* elements that are observed from isofemale lines can also be observed in pool-seq samples from the same populations. Our work supports the view that genomic *P* element content alone is not sufficient to explain variation in gonadal dysgenesis across strains of *D. melanogaster*, and informs future efforts to decode the genomic basis of geographic and temporal differences in *P* element induced phenotypes.

## Introduction

A substantial portion of eukaryotic genomes is represented by transposable elements (TEs). These TE families include those that colonized genomes long ago during the evolution of the host species and groups, but also those that have appeared in their host genomes recently. One of the best examples of a newly acquired TE is the *P* element in *Drosophila melanogaster* which is thought to have been acquired at least 70 years ago as a result of a horizontal transmission event from *D. willistoni* (Anxolabéhère, Kidwell & Periquet, 1988; Daniels et al., 1990), a species that inhabits South America, the Caribbean, and southern parts of North America. Laboratory strains of *D. melanogaster* established from wild populations before the 1950s did not contain *P* element, while by the late 1970s this TE family was found in all natural populations worldwide (Anxolabéhère, Kidwell & Periquet, 1988).

Classical work has shown that the presence of *P* elements induces a number of phenotypes in *D. melnaogaster* that can be characterized by the so-called “P-M hybrid dysgenesis” assay (Kidwell, Kidwell & Sved, 1977). Among the most prominent *P* element induced phenotypes is gonadal dysgenesis (GD), which is the key marker determining P-M status in particular strains of flies (Kidwell, Kidwell & Sved, 1977; Engels & Preston, 1980). In the P-M system, fly strains can be categorized as follows: P-strains have the ability to activate and repress *P* element transposition, P’-strains only have the ability to activate *P* element transposition, Q-strains only have the ability to repress *P* element transposition, and M-strains have neither the ability to activate or repress *P* element transposition (Kidwell, Kidwell & Sved, 1977; Engels & Preston, 1980; Anxolabéhère & Quesneville, 1998). M-strains that carry *P* element sequences in their genome are called M’-strains, while true M-strains are completely devoid of *P* elements (Bingham, Kidwell & Rubin, 1982). GD phenotypes were originally proposed to be mediated by repressor proteins encoded by full-length *P* elements or internally-truncated *P* elements that prevent *P* element transposition and subsequent DNA damage (Rio, 2002). Other work posits that these phenotypes mostly arise due to RNAi-based repression mediated by piRNAs produced by telomeric *P* elements whose effects are amplified by RNAs produced from other *P* elements (Ronsseray, Lehmann & Anxolabehere, 1991; Brennecke et al., 2007; Simmons et al., 2014; Simmons et al., 2015). More recently, some authors have questioned the classical view that GD phenotypes are caused solely by *P* elements or whether other factors may be involved (Zakharenko & Ignatenko, 2014; Ignatenko et al., 2015).

To better understand *P* element invasion dynamics and the molecular mechanisms that underlie P-M hybrid dysgenesis, many studies have surveyed variation in GD phenotypes across natural populations of *D. melanogaster* (Kidwell, Frydryk & Novy, 1983; Anxolabéhère et al., 1985; Anxolabéhère et al., 1988; Boussy & Kidwell, 1987; Anxolabéhère, Kidwell & Periquet, 1988; Boussy et al., 1988; Gamo et al., 1990; Matsuura et al., 1993; Itoh et al., 1999; Itoh et al., 2001; Itoh et al., 2004; Itoh et al., 2007; Bonnivard & Higuet, 1999; Ogura et al., 2007; Onder & Bozcuk, 2012; Onder & Kasap, 2014; Ignatenko et al., 2015). These studies reveal that most natural strains of *D. melanogaster* are P, Q, or M’, but that there can be substantial variation in the frequency of GD phenotypes within and between populations. In addition, variation among populations in GD phenotypes is thought to be relatively stable since their initial transitions from M cytotype to P, Q and M’ cytotypes (Gamo et al., 1990; Matsuura et al., 1993; Boussy et al., 1998; Bonnivard & Higuet, 1999; Itoh et al., 2001; Itoh et al., 2004; Itoh et al., 2007; Ogura et al., 2007). For example, Australian populations demonstrate a north-south cline of the frequency of various GD phenotypes (Boussy & Kidwell, 1987), which underwent only minor changes in the frequencies of truncated and full-size copies of the *P* element a decade later (Ogura et al., 2007).

A number of studies have also used Southern blotting, *in situ* hybridization to polytene chromosomes, or PCR to understand how the genomic composition of *P* elements varies in relation to GD phenotypes (Bingham, Kidwell & Rubin, 1982; Todo et al., 1984; Engels, 1984; Boussy et al., 1988; Itoh et al., 1999; Itoh et al., 2001; Itoh et al., 2007; Itoh & Boussy, 2002; Ruiz & Carareto, 2003; Onder & Kasap, 2014; Ignatenko et al., 2015). These studies have revealed that, irrespective of GD phenotype, the majority of *D. melanogaster* strains harbor multiple copies of full-length *P* elements (*FP*) along with multiple copies of the truncated repressor element known as “*KP*” (Black et al., 1987), suggesting a complex relationship between the presence of different types of *P* elements in a genome and GD phenotypes. Attempts to quantify the relationship between absolute *P* element copy number or *KP*/*FP* ratios and GD phenotypes have revealed weak or no correlations between genomic *P* element composition and GD phenotypes (Todo et al., 1984; Engels, 1984; Boussy et al., 1988; Ronsseray, Lehmann & Anxolabéhère, 1989; Rasmusson et al., 1990; Itoh et al., 1999; Itoh et al., 2004; Itoh et al., 2007; Bonnivard & Higuet, 1999; Itoh & Boussy, 2002). However, these conclusions rely on estimates of *P* element copy number based on low-resolution hybridization data.

The recent widespread availability of whole-genome shotgun sequences for *D. melanogaster* and related species offers the possibility of new insights into the relationship between *P* element genomic content and GD phenotypes with unprecedented resolution. Recently, Hill, Schlötterer & Betancourt (2016) showed that *P* element copy number estimated from 12 lines from a single N. American population of *D. simulans* are weakly associated with GD phenotypes. Likewise, Srivastav & Kelleher (2017) investigated a panel of 33 inbred lines from a single N. American population of *D. melanogaster* and found weak or no association between various measures of *P* element content based on whole genome sequence data and GD phenotypes. These pioneering genomic studies show the promise of using whole genome data to investigate how *P* element genomic content potentially correlates with P-M dysgenesis across natural populations. To date, hundreds of samples of *D. melanogaster* genomes from populations around the world have been sequenced that can be freely used for population and genomic analyses (Mackay et al., 2012; Pool et al., 2012; Bergman & Haddrill, 2015; Grenier et al., 2015; Lack et al., 2016). Moreover, a growing number of computational algorithms have been designed for *de novo* TE insertion discovery, annotation, and population analysis in *Drosophila* (Kofler, Betancourt & Schlötterer, 2012; Linheiro & Bergman, 2012; Cridland et al., 2013; Nakagome et al., 2014; Zhuang et al., 2014; Rahman et al., 2015). However, comparison of different methods for detecting TEs in *Drosophila* NGS data has shown that they identify different subsets of TE insertions (Song et al., 2014; Rahman et al., 2015), and thus determining which TE detection method is best for specific biological applications remains an area of active research (Ewing, 2015; Rishishwar, Marino-Ramirez & Jordan, 2016; Nelson, Linheiro & Bergman, 2017).

To better understand the molecular basis of differences in cytotype status among populations, we investigated the relationship between *P* element content and GD phenotypes in whole genome shotgun sequences from three worldwide populations of *D. melanogaster*. By combining previously published GD assay data (Ignatenko et al., 2015) with estimates of *P* element content (this study) based on genomic data of the same strains (Bergman & Haddrill, 2015), we show that populations can differ significantly in their *P* element content, yet show similar distributions of GD phenotypes. Furthermore, we show that *P* element content is not correlated with the degree of a GD phenotype exhibited by a strain. We also investigate several bioinformatics strategies for detecting *P* elements in strain-specific and pooled genomic data to ensure robustness of our conclusions and help guide further genomic analysis. Our work supports previous conclusions that *P* element content alone is not sufficient to explain variation in GD phenotypes, and informs future efforts to decode the genomic basis of differences in *P* element induced phenotypes over time and space.

## Materials and Methods

### Estimation of *P* element content in isofemale strain and pool-seq genomic samples

Whole genome shotgun sequences for *D. melanogaster* from three geographic regions—N. America (Athens, Georgia, USA), Europe (Montpellier, France), and Africa (Accra, Ghana), described in Verspoor & Haddrill (2011)—were downloaded from the European Nucleotide Archive (ERP009059) (Bergman & Haddrill, 2015). These genomic data were collected from adult females using a uniform library preparation and sequencing strategy (thus mitigating many possible technical artifacts) and include data from both individual isofemale strains and pools of single flies derived from isofemale strains (see (Bergman & Haddrill, 2015) for details). The median insert size of libraries analyzed in this study ranged from 453–490 bp with a mean (±S.D.) of 475  ± 8 bp. The mean depth of coverage of the libraries analyzed in this study ranged from 48×–56× with a mean (±S.D.) of 53 ×  ± 1.8. In total, 50 isofemale strain genomes were analyzed: N. America (*n* = 15), Europe (*n* = 20), and Africa (*n* = 15). Two pool-seq samples were analyzed for each population (N. America (15 and 30 strains), Europe (20 and 39 strains), and Africa (15 and 32 strains)). The smaller pools for each population include one individual from the same isofemale strains that were sequenced as strain-specific samples from that population; the larger pools contain one individual from the same strains as the smaller pools, plus individuals from additional isofemale strains from the same population that were not sequenced as strain-specific samples.

Estimates of the number of *P* elements per sample were generated using two different non-reference TE detection methods (TEMP (Zhuang et al., 2014) and RetroSeq (Keane, Wong & Adams, 2013)), as well as by computing coverage of the *P* element normalized to the *D. melanogaster* reference genome. Non-reference TE detection methods like TEMP and RetroSeq can identify individual insertion sites, but cannot differentiate full-length from truncated copies and have detection biases that are not fully characterized. Therefore, normalized coverage across diagnostic sections of the *P* element consensus sequence was used to provide a complementary estimate of copy number and to estimate the abundance of full-length and truncated *P* element copies.

TEMP (revision d2500b904e2020d6a1075347b398525ede5feae1) and RetroSeq (revision 700d4f76a3b996686652866f2b81fefc6f0241e0) were run using the McClintock pipeline (revision 3ef173049360d99aaf7d13233f9d663691c73935; Nelson, Linheiro & Bergman, 2017). McClintock was run across the major chromosome arms (chr2L, chr2R, chr3L, chr3R, chr4, chrY, and chrX) of the UCSC dm6 version of the Release 6 reference genome (Hoskins et al., 2015) using the following options: -C -m “retroseq temp” -i -p 12 -b. We note that TEMP and RetroSeq both base their predictions on a common BAM file generated by the McClintock pipeline, and thus read mapping is controlled for in our analysis. Reference TE annotations needed for TEMP were generated automatically by McClintock using RepeatMasker (version open-4.0.6). The *D. melanogaster* TE library used by McClintock to predict reference and non-reference TE insertions is a slightly modified version of the Berkeley *Drosophila* Genome Project TE data set v9.4.1 (https://github.com/cbergman/transposons/blob/master/misc/D_mel_transposon_sequence_set.fa; described in Sackton et al., 2009).

In addition to providing “raw” output for each component method in the standardized zero-based BED6 format, McClintock generates “filtered” output tailored for each method (Nelson, Linheiro & Bergman, 2017). McClintock filters TEMP output to: (i) eliminate predictions where the start or end coordinates had negative values; (ii) retain predictions where there is sequence evidence supporting both ends of an insertion; and (iii) retain predictions that have a ratio of reads supporting the insertion to non-supporting reads of >1/10. Likewise, McClintock filters RetroSeq output to: (i) eliminate predictions where two different TE families shared the same coordinates; and (ii) retain predictions assigned a call status of greater than or equal to six as defined by Keane, Wong & Adams (2013).

Isofemale strain genomes from these populations have been reported to contain residual heterozygosity (Lack et al., 2016), presumably due to incomplete inbreeding in these strains prior to genome sequencing. To control for potential incomplete inbreeding in strain-specific sequences, we also generated estimates of *P* element abundance based on the sample-frequency weighted-sum of TEMP predictions. Specifically, we weighted each *P* element prediction in the raw or filtered TEMP BED files by the frequency of that prediction in the original TEMP insertion.refined.bp.summary output file for that sample. Frequency weighted estimates could not be generated for RetroSeq since this method does not provide an estimate of sample frequency for non-reference TE predictions.

Normalized *P* element coverage was computed with SAMtools (v.1.4.1) (Li et al., 2009) using the BAM file generated by McClintock that was used for TEMP and RetroSeq predictions above. The RepeatMasker annotation of TEs in the dm6 reference genome generated by McClintock was used to generate a complementary BED file of unique regions in the major chromosome arms (chr2L, chr2R, chr3L, chr3R, chr4, chrY, and chrX). SAMtools depth was then used to calculate the mean coverage of the unique regions of the dm6 genome and across the canonical *P* element sequence. Mean coverage per site was then averaged across the length of *P* element exon 0 (positions 153–442) and exon 2 (positions 1222–1947), and the resulting value was divided by mean coverage of the unique regions of the dm6 genome to generate normalized coverage of each exon. Normalized coverage of exon 0 was used to estimate overall *P* element copy number. Normalized coverage of exon 2 was used to estimate the number of full-length *P* elements (*FP*). Normalized coverage of exon 0 minus exon 2 was used to estimate the number of truncated *P* elements that are predominantly of the *KP* type (Black et al., 1987). Coverage profiles across the entire *P* element sequence were plotted as the average [±95% C.I.] across all isofemale lines and as the loess smoothed (span  = 0.01) values for pool-seq samples.

### Gonadal dysgenesis phenotypes

We re-analyzed GD assay data from Ignatenko et al. (2015) for 43 of the 50 isofemale strains analyzed for genomic *P* element content above. Definitions of A and A* crosses in Ignatenko et al. (2015) are inverted relative to those proposed by Engels & Preston (1980), and were standardized prior to re-analysis here. Cross A measures the activity of tester strain males mated to M-strain Canton-S females; cross A* measures the susceptibility of tester females mated to a P-strain Harwich males. P, P′, Q, and M-strains were defined according to Kidwell, Frydryk & Novy (1983) and Anxolabéhère & Quesneville (1998).

Graphical and statistical analyses were performed in the R programming environment (version 3.4.0).

## Results

### *P* element content varies among isofemale strains from N. America, Europe and Africa

We initially generated estimates of *P* element abundance in the genomes of 50 isofemale strains from three worldwide populations using two non-reference TE detection methods: TEMP (Zhuang et al., 2014) and RetroSeq (Keane, Wong & Adams, 2013) (Files S1, S2). TEMP and RetroSeq were chosen for *P* element detection because they showed the highest performance to accurately detect non-reference TE insertions in a previous simulation study (Nelson, Linheiro & Bergman, 2017). We also generated estimates of *P* element abundance using a complementary approach based on coverage of reads mapped to the canonical *P* element sequence normalized to coverage of the *D. melanogaster* reference genome (File S1). To account for potential false positive predictions, we also investigated the effects of the default filtering of TEMP and RetroSeq output performed by McClintock (Nelson, Linheiro & Bergman, 2017), a meta-pipeline that runs and parses multiple TE insertion detection methods. Additionally, to deal with potential residual heterozygosity in strain-specific genome sequences, we investigated weighting TEMP predictions by their sample frequency. We note that neither TEMP nor RetroSeq can differentiate full-length from truncated insertions in their output, and conversely normalized coverage cannot resolve the location of individual insertions. Moreover, we note that heterochromatic contigs were omitted from TEMP and RetroSeq analysis, however heterochromatic insertions can contribute to estimates of *P* element content based on coverage.

**Figure 1:**
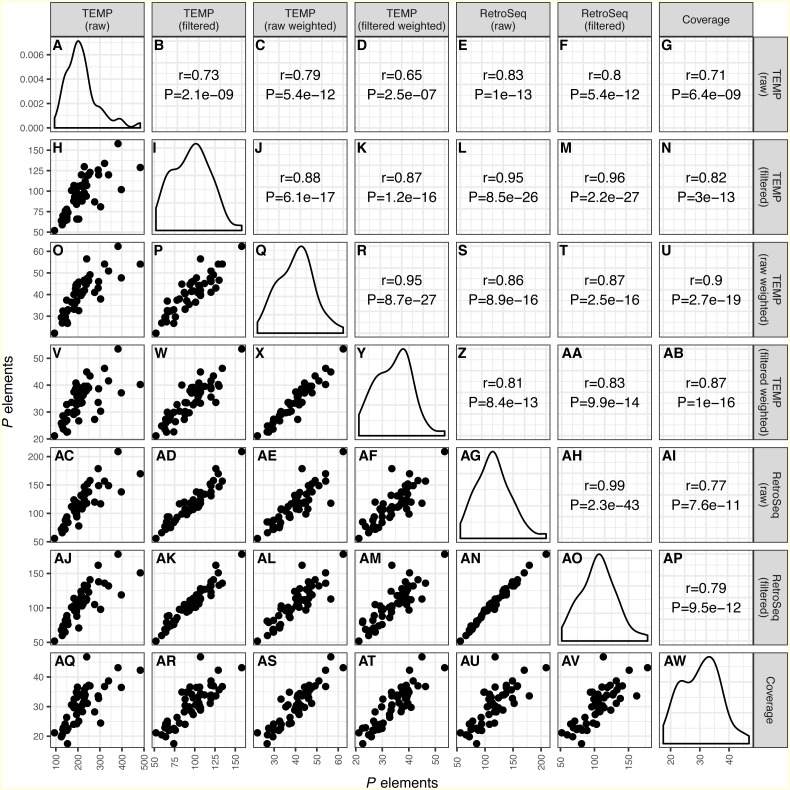
Correlation across different bioinformatics methods in numbers of predicted *P* elements for a worldwide sample of *D. melanogaster* isofemale strains from N. America, Europe and Africa. Numbers of *P* elements predicted by TEMP or RetroSeq shown are before (raw) and after (filtered) filtering by McClintock, and for TEMP are also weighted by sample frequency. *P* element numbers based on coverage are estimated by normalizing coverage in exon 0 of the *P* element canonical sequence to the coverage of the unique regions of the major arms of the *D. melanogaster* genome. Each dot in H, O-P, V-X, AC-AF, AJ-AN, AQ-AV represents a single isofemale strain. Histograms in panels A, I, Q, Y, AG, AO, AW represent the distribution of the estimated number of *P* elements per strain. Numbers in B-G, J-N, R-U, Z-AB, AH-AI, AP represent the Pearson correlation coefficients and associated *P*-values for correlations between pairs of methods. Note that the scales on the *x*-axis and *y*-axis vary for each method.

As shown recently in related work by Srivastav & Kelleher (2017), we find that overall numbers of *P* elements predicted in strain-specific genome sequences were well correlated across all bioinformatics approaches investigated (*r* ≥ 0.65; *P* < 2.5e − 7) (Fig. 1). The lowest correlation between distinct methods was for raw TEMP predictions and normalized coverage (*r* = 0.71) (Figs. 1G and 1AQ), and the highest correlation was for the filtered TEMP and filtered RetroSeq datasets (*r* = 0.96) (Figs. 1M and 1AK). McClintock filtering reduced the numbers of predicted *P* elements per strain substantially for TEMP but less so for RetroSeq (Fig. 1, Table 1) and generally increased the correlation of TEMP and RetroSeq predictions with other methods (Fig. 1). These results suggest that all bioinformatic approaches investigated capture similar aspects of variation in *P* element abundance across strains, and that filtering by McClintock improves the consistency of TE predictions made by TEMP and RetroSeq on isofemale strains.

**Table 1:** Average numbers (±S.D.) of *P* elements estimated by TEMP, RetroSeq and normalized coverage in isofemale strains from three worldwide populations of *D. melanogaster*. Columns labeled raw and filtered represent output generated by each method before or after default filtering by McClintock, respectively, and columns labeled weighted represent the sum of the number of insertions weighted by sample frequency (see Materials and Methods for details).

Population	# strains	TEMP raw	TEMP filtered	TEMP raw weighted	TEMP filtered weighted	RetroSeq raw	RetroSeq filtered	Coverage
N. America	15	159.3 ± 48.5	68.7 ± 8.9	30.7 ± 4.1	26.4 ± 2.9	83.4 ± 15.7	77.0 ± 13.8	22.4 ± 2.0
Europe	20	247.3 ± 78.5	106.7 ± 13.9	43.3 ± 6.0	35.9 ± 4.3	127.0 ± 22.1	116.5 ± 18.3	34.4 ± 4.6
Africa	15	233.7 ± 60.1	108.3 ± 20.2	45.8 ± 6.2	39.9 ± 5.0	131.9 ± 29.3	120.5 ± 22.2	32.9 ± 4.4

Despite providing correlated estimates of abundance across strains, the absolute numbers of estimated *P* elements in these strain-specific genome data differed substantially across methods (Table 1). Raw output from TEMP and RetroSeq indicate that flies from these isofemale lines contain ∼80–250 *P* element insertions, whereas McClintock filtered predictions for TEMP and RetroSeq suggest they contain ∼70–120 *P* element insertions. In contrast, estimates of abundance based on frequency-weighted TEMP predictions and normalized coverage indicates that flies from these samples only contain ∼20–45 *P* elements per haploid genome, with weighted TEMP predictions giving slightly higher estimates of *P* element abundance than coverage. Previous results based on Southern blotting suggest 30–50 *P* elements per haploid genome (Bingham, Kidwell & Rubin, 1982; Ronsseray, Lehmann & Anxolabéhère, 1989; Bonnivard & Higuet, 1999; Itoh & Boussy, 2002). We interpret the discrepancy between unweighted non-reference TE detection methods and either weighted TEMP or normalized coverage, together with the similarity between the latter approaches and Southern hybridization data, as being consistent with overestimation by unweighted non-reference TE detection methods due to residual heterozygosity stemming from incomplete inbreeding in these strains (Lack et al., 2016). If multiple haplotypes persist at many loci in these strains due to partial inbreeding, this could elevate the number of insertion sites per strain predicted by unweighted non-reference TE detection methods (relative to the number expected from a haploid genome), but not increase estimated copy number based on weighted non-reference TE detection methods or normalized coverage (because polymorphic insertion sites would only contribute to abundance for these methods in a manner that is proportional to their frequency). The relatively large decrease in the number of predictions observed in raw versus filtered output for TEMP but not RetroSeq is also consistent with this interpretation since TEMP (but not RetroSeq) is capable of identifying polymorphic insertions in a genomic sample. Since estimates of *P* element abundance based on weighted TEMP predictions and coverage are concordant with independent estimates based on Southern hybridization, we conclude that these methods provide more accurate estimates of *P* element abundance per strain and therefore focused further analysis on these two approaches.

**Figure 2:**
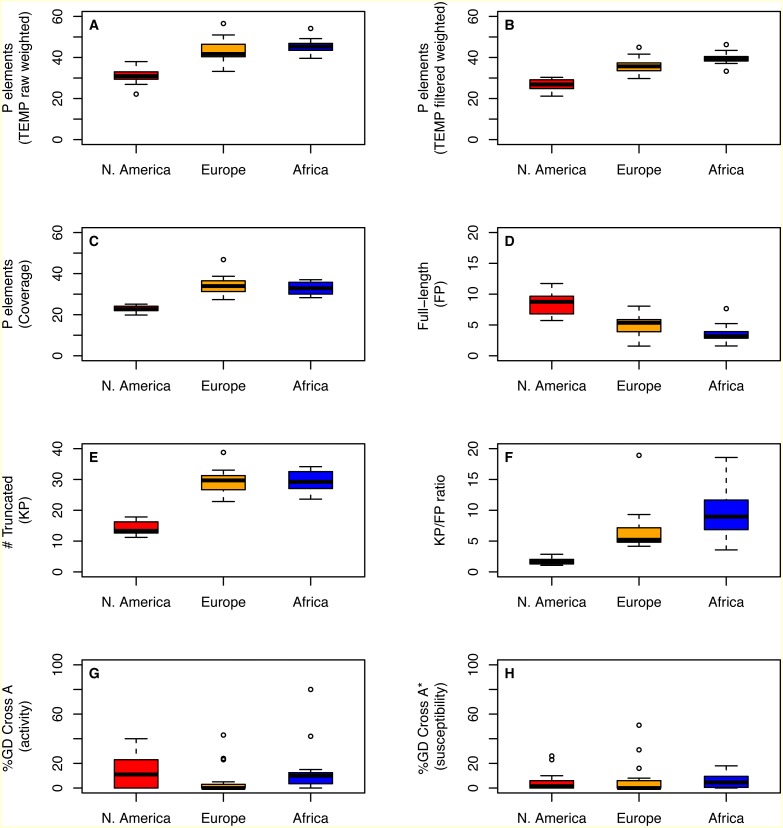
Distribution of *P* element content and GD phenotypes for isofemale strains within and between three worldwide populations of *D. melanogaster*. Distributions for total number of predicted *P* elements, full-length *P* elements (*FP*), ratio of truncated to full-length elements (*KP*∕*FP* ratio), and %GD in A and A* crosses are shown as boxplots with black lines representing median values, boxes representing the interquartile range (IQR), whiskers representing the limits of values for strains that lie within 1.5 × IQR of the upper or lower quartiles, and circles representing strains that lie outside 1.5 × IQR of the upper or lower quartiles. GD data are taken from Ignatenko et al. (2015) and were standardized to definitions proposed by Engels & Preston (1980) prior to re-analysis here. Numbers of *P* elements predicted by TEMP shown are before (raw) and after (filtered) standard filtering by McClintock and are weighted by sample frequency. Analogous results for unweighted output of TEMP or RetroSeq are shown in Fig. S1.

Despite differences in how they capture aspects of TE abundance, estimates of *P* element abundance based on weighted TEMP predictions or coverage show significant differences in the number of *P* elements per strain across populations (One-way ANOVA; *F* > 35.27; 2 *d.f.*, *P* < 1.3e − 9), with strains from the N. American population showing a clear reduction in *P* element abundance relative to the European and African populations (Table 1; Figs. 2A–2C). Similar results are observed for abundance estimates based on unweighted TEMP and RetroSeq predictions (One-way ANOVA; *F* > 9.26; 2 *d.f.*, *P* < 5e−4) (Fig. S1). Moreover, by contrasting normalized coverage in exon 0 versus exon 2 of the *P* element to estimate numbers of full-length (*FP*) and truncated (*KP*) copies, we found clear differences among populations in the number of full-length (One-way ANOVA; *F* = 32.4; 2 *d.f.*, *P* = 4.33e−9) and truncated (One-way ANOVA; *F* = 102.1; 2 *d.f.*, *P* < 2e−16) copies, with slightly more full-length and many fewer truncated copies in the N. American population relative to the European and African populations (Figs. 2D–2E). Together, this variation in *P* element structure leads to a strong difference in the *KP*∕*FP* ratio across populations (One-way ANOVA; *F* = 20.0; 2 *d.f.*, *P* < 9.52e−7) (Fig. 2F).

**Figure 3:**
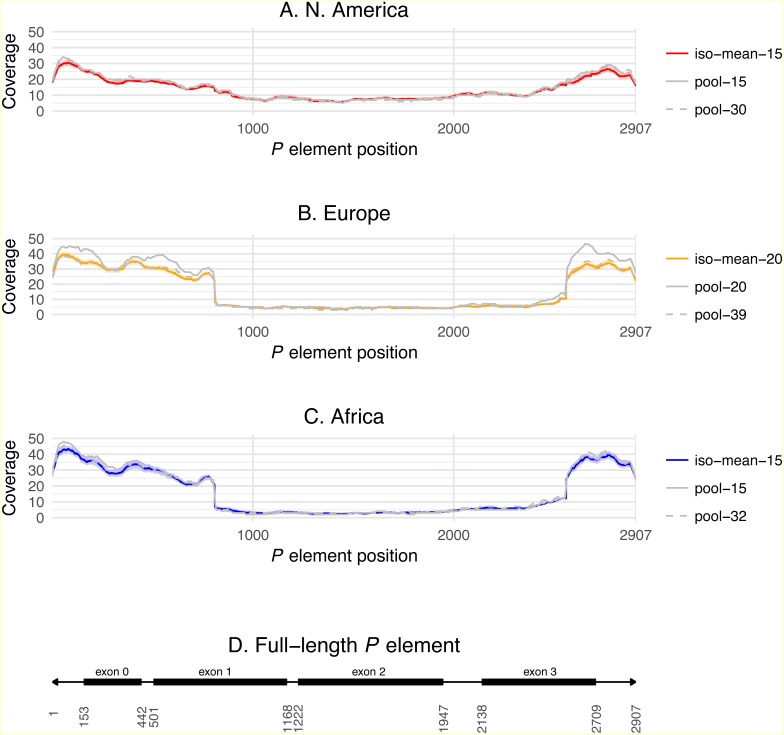
*P* element coverage profiles for isofemale lines and pool-seq samples from three worldwide populations of *D. melanogaster*. (A–C) show the depth of coverage at each position in the canonical *P* element for the mean of isofemale lines (dark color) and 95% C.I. of isofemale lines (light color), smaller pool-seq sample with the same strains as the isofemale data (solid grey), and larger pool-seq sample with the same strains as the isofemale data plus additional strains (dashed grey). Pool-seq profiles were loess smoothed (span = 0.01). (D) shows the structure of a canonical *P* element with exons 0–3 shown as black boxes and the 31 bp terminal inverted repeats shown as arrowheads. Average coverage across exon 0 (positions 153–442) and exon 2 (positions 1222–1947) are used to estimate the number of total, full-length and truncated *P* elements in each sample.

Differences across populations in overall *P* element abundance and the relative abundance of full-length and truncated copies can be visualized by plotting normalized coverage averaged across isofemale strains over the length of the *P* element canonical sequence (Fig. 3). Relative to European and African strains, N. American strains have lower coverage at the termini of the *P* element including exon 0 (positions 153–442, present in truncated and full-length copies), but slightly higher coverage in exon 2 (positions 1222–1947, present only in full-length copies). European and African strains also have a steep changes in coverage precisely at the breakpoints expected for the *KP* element (positions 807 and 2561), which are not observed in N. American strains. Together, these results demonstrate clear differences in *P* element abundance and structure among isofemale strains from N. American versus European and African populations. Additionally, these coverage profiles reveal positional variation in depth across exon 0 in all populations, which may explain the slightly lower abundance estimates obtained from coverage relative to weighted TEMP predictions.

### Lack of association between *P* element content and gonadal dysgenesis phenotypes

To address whether the variation in genomic *P* element content that is observed across populations affects geographic variation in hybrid dygenesis, we compared *P* element abundance from 43 isofemale strains analyzed above with previously-published GD phenotypes for the same strains (Ignatenko et al., 2015). As reported in Ignatenko et al. (2015), isofemale strains from these populations were mainly P, M and Q (Table 2, Fig. S2). Based on our genomic analysis (File S1), all strains in these populations that are defined phenotypically as M are actually M’. For N. American and African populations, the degree of *P* element activity tends to vary more across strains within populations relative to susceptibility (Table 2, Figs. 2G–2H). However, we found no evidence for systematic differences across populations in the degree of activity (One-way ANOVA; *F* = 0.06, 2 *d.f.*, *P* = 0.94) or susceptibility (One-way ANOVA; *F* = 1.66, 2 *d.f.*, *P* = 0.2). Taken together with the genomic analysis above, these results suggest that population-level differences in the abundance or structure of *P* elements do not necessarily translate into differences in the frequency of GD phenotypes across populations.

**Table 2:** Gonadal dysgenesis (GD) levels and P-M status for isofemale strains of *D. melanogaster* obtained from natural populations in N. America, Europe and Africa. %GD for cross A (tester strain males versus M-strain Canton-S females) and cross A* (P-strain Harwich males versus tester strain females) are based on data reported in Ignatenko et al. (2015). Cross A and A* labels in Ignatenko et al. (2015) are inverted relative to those proposed by Engels & Preston (1980) and were converted to standard labels prior to analysis here. P-M status for individual strains is according to Ignatenko et al. (2015). Phenotypically M-strains are in fact M’-strains based on analysis of genomic data (Files S1, S2).

Population	Year of collection	Cross A (%GD ± SD)	Cross A* (%GD ± SD)	# strains	M	Q	P′	P
N. America	2009	13.4 ± 12.3	5.4 ± 8.6	14	3	4	0	7
Europe	2010	6.1 ± 12.2	6.8 ± 14.0	17	2	12	1	2
Africa	2010	16.3 ± 22.8	6.0 ± 5.9	12	1	8	1	2
Total				43	6	24	2	11

**Figure 4:**
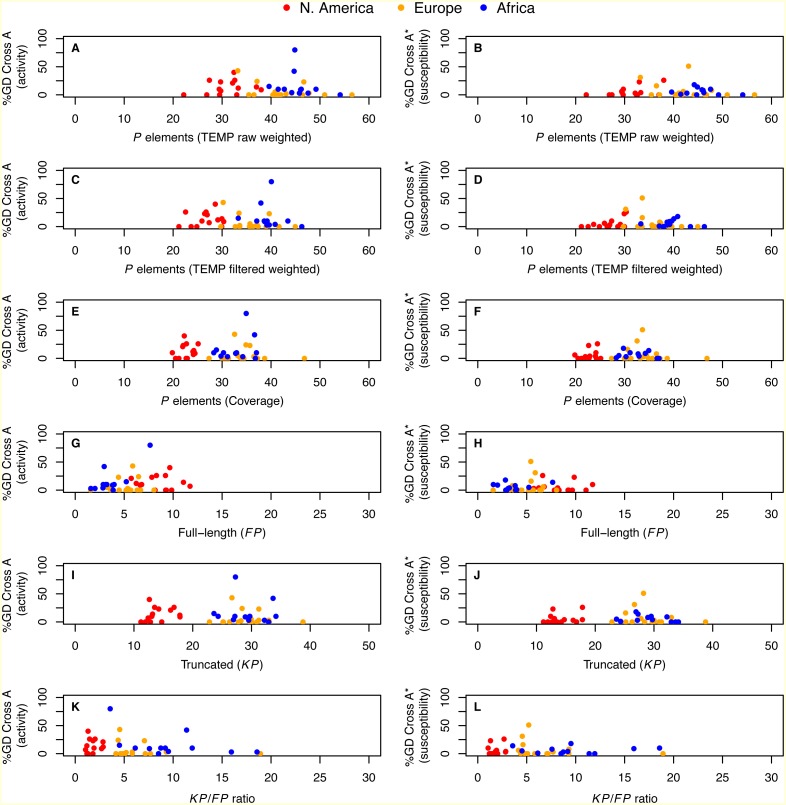
Relationship between *P* element content and GD phenotypes in worldwide sample of *D. melanogaster* isofemale strains from N. America, Europe and Africa. %GD data are from Ignatenko et al. (2015) and use the same standardized definitions as in Fig. 2. Numbers of *P* elements predicted by TEMP shown are before (raw) and after (filtered) standard filtering by McClintock and are weighted by sample frequency. Analogous results for unweighted output of TEMP or RetroSeq are shown in Fig. S3. Each dot represents an isofemale strain.

Integrating our genomic predictions with these published GD data at the level of individual strains, we directly tested whether the number of *P* elements per strain is associated with either activity or susceptibility phenotypes in GD assays. We found that neither the degree of activity nor the degree of susceptibility was significantly correlated with *P* element abundance estimated from weighted TEMP predictions or coverage (*P* > 0.11; Fig. 4). Similar results were obtained using the unweighted output of TEMP and RetroSeq as well (*P* > 0.11; Fig. S3). Generalized linear models with Gaussian errors incorporating population as a factor also failed to identify significant associations between any measure of *P* element abundance and GD phenotypes when corrected for multiple testing (*P* > 0.038). These results suggest that there is no simple relationship between the total abundance or relative abundance of full-length versus truncated *P* elements and GD phenotypes at the level of individual strains, confirming conclusions inferred from the population level above.

### Population difference in *P* element content can be observed in pool-seq samples

Pooled-strain sequencing (pool-seq) is a cost-effective strategy to sample genomic variation across large numbers of strains and populations (Schlotterer et al., 2014). However, little empirical evidence exists reporting how biological conclusions derived from pool-seq data relate to those from strain-specific sequencing for the same samples or populations. The populations analyzed here provide a unique opportunity to address such questions since pool-seq samples are available for the same set of isofemale strains with strain-specific genomic data. Two pool-seq samples are available for each population that differ in the number of individuals (one per isofemale strain) used: N. America (*n* = 15 and *n* = 30), Europe (*n* = 20 and *n* = 39), and Africa (*n* = 15 and *n* = 32). The smaller pools for each population each include one individual from the same isofemale strains that were sequenced as strain-specific samples from that population; the larger pools contain one individual from the same strains as the smaller pools, plus individuals from additional isofemale strains from the same population that were not sequenced as strain-specific samples. Thus, the smaller pool-seq samples are a nested subset of the larger pool-seq samples, and pool-seq samples from the same population are not fully independent from one another.

To address whether the differences among populations we observed in the number of *P* elements observed in isofemale strain data can also be detected using different bioinformatics approaches in pool-seq data, we estimated the number of *P* elements in pool-seq samples from the same populations using weighted TEMP predictions and coverage (Table 3). Because depth of coverage per strain in pool-seq samples is inversely proportional to the number of strains in the pool, estimates of *P* element content based on weighted TEMP predictions or normalized coverage offer the most straightforward approach to compare strain-specific and pool-seq results. As expected if pool-seq samples accurately reflect the average based on strain-specific sequencing, estimates of *P* element abundance in pool-seq samples from raw weighted TEMP predictions and coverage (Table 3) are similar to those derived from strain-specific sequencing of the same population (Table 1). For all pool-seq samples, however, estimates of the number of *P* elements per strain based on raw weighted TEMP predictions or coverage are slightly higher than the average weighted TEMP predictions based on isofemale strain data. In contrast, estimates of *P* element abundance based on filtered weighted TEMP predictions in pool-seq data are much lower than other methods applied to pool-seq data or for filtered weighted TEMP predictions on strain-specific genomic data, especially for the larger pools. Underestimation of *P* element abundance from filtered weighted TEMP predictions presumably arises because many real *P* element insertions in these pool-seq samples exist at a frequency lower than the cutoff of 1/10 required by McClintock filtering. This interpretation is consistent with the fact that the vast majority of non-redundant *P* element insertion sites in strain-specific genomic data are found only in a single strain (>75%), and these singleton insertions would therefore be at a frequency lower than 1/10 in any of the strain pools.

**Table 3:** Estimated number of *P* elements identified by TEMP and normalized coverage in pool-seq samples from three worldwide populations of *D. melanogaster*. Columns for TEMP labeled raw and filtered represent output generated before or after default filtering by McClintock, respectively, and data shown are the sum of the number of insertions weighted by sample frequency (see Materials and Methods for details).

Population	# strains	TEMP raw weighted	TEMP filtered weighted	Coverage
N. America	15	36.2	7.3	22.8
N. America	30	37.5	5.4	23.0
Europe	20	53.1	14.1	37.8
Europe	39	47.4	7.4	34.8
Africa	15	54.6	15.9	35.6
Africa	32	53.3	5	33.8

Importantly, estimates of *P* element abundance based on raw weighted TEMP predictions or coverage in N. America pool-seq samples show a clear reduction in the estimated number of *P* elements relative to European and African pool-seq samples (Table 3), consistent with the statistically significant differences among populations observed using samples of strain-specific genomic data above. Furthermore pool-seq samples qualitatively and quantitatively (with the exception of the pool-20 sample in Europe) recover population differences in depth of coverage profiles across the canonical *P* element sequence (Fig. 3). These conclusions are true both for the smaller pools that precisely match the strain-specific samples, as well as the larger pools that include genomes not sampled in the strain-specific data. These results suggest that frequency-weighted raw TEMP predictions and coverage are viable methods for detecting differences in *P* element abundance and structure across *D. melanogaster* populations using pool-seq data.

## Discussion

Here we performed a detailed analysis of *P* element content in genomes of isofemale strains and pool-seq samples from three worldwide populations of *D. melanogaster* with published GD phenotypes. Our results allowed us to draw several conclusions about the detection of *P* elements in *D. melanogaster* short-read shotgun sequence data that can be used to inform future efforts to decode the genomic basis of GD phenotypes.

For samples derived from isofemale strains, we find that multiple approaches to estimate *P* element abundance based on non-reference TE predictions or depth of coverage generate well-correlated numbers of *P* element predictions per strain (Fig. 1) (see also Srivastav & Kelleher, 2017). In the isofemale strain genomes analyzed here, we find that non-reference TE detection methods that do not account for the frequency of the predicted TE in the sample generate much higher than expected numbers of *P* elements relative to estimates based on frequency-weighted TEMP predictions or depth of coverage (Fig. 1, Table 1). Previous results based on SNP analysis suggest that the isofemale strain genomes analyzed here have residual heterozygosity due to incomplete inbreeding (Lack et al., 2016). The discrepancy between estimates of *P* element abundance based on unweighted TEMP or RetroSeq predictions and weighted TEMP predictions or coverage, together with the similarity between weighted TEMP predictions or coverage estimates and independent estimates based on Southern hybridization data, support the interpretation that residual heterozygosity due to incomplete inbreeding causes an excess of unweighted TEMP and RetroSeq predictions in these strain-specific genomic data. Further support for this interpretation comes from the fact that weighted and unweighted TEMP predictions did not show striking differences in average *P* element abundance in a recent study using the highly-inbred *Drosophila* Genetic Reference Panel (DGRP) strains (Srivastav & Kelleher, 2017). These results suggest that efforts to quantify TE abundance using *D. melanogaster* isofemale strain genomic data should control for incomplete inbreeding using methods that take sample frequency into consideration (such as TEMP or TIDAL Rahman et al., 2015).

Regardless of prediction method, we observed clear differences in the abundance and structure of *P* elements among isofemale strains obtained from these populations (Figs. 2 and 3, Fig. S1). Specifically, strains from the N. American population had the fewest *P* elements but the highest proportion of full-length elements. Evidence for fewer *P* elements and a higher proportion of complete elements in the N. American population could also be detected in pool-seq samples using coverage-based methods (Fig. 3, Table 3). These results are somewhat unexpected given that the *P* element is thought to have first been horizontally transferred into a N. American population before invading the rest of the world (Anxolabéhère, Kidwell & Periquet, 1988). If the accepted scenario of worldwide spread for the *D. melanogaster P* element is true, our results suggest that it may not always be possible to interpret the observation that populations with fewer *P* elements or a higher proportion of full-length *P* elements have more recently-established *P* element infections, as previously done in *D. simulans* (Kofler et al., 2015). Moreover, our observations suggest that N. American populations may exert copy number control over *P* elements, especially over truncated *KP*-like elements, in a manner that differs from other populations. This could be due to the presence of one of more full-length *P* element insertions in the N. American population capable of exerting strong repression in *trans*, similar to that shown for *P* elements at the 1A insertion site at the tip of the X-chromosome (Ronsseray, Lehmann & Anxolabehere, 1991), that are not present in the European or African populations.

Despite finding strong evidence for population-level differences in *P* element content, we found no evidence for differences in GD phenotypes across populations. Furthermore, we found no simple linear relationship between *P* element content based on genomic data and the strength of GD phenotypes across isofemale strains (Fig. 4, Fig. S3). Our results are consistent with recent findings based on genomic data (Hill, Schlötterer & Betancourt, 2016; Srivastav & Kelleher, 2017) and classical attempts to connect total numbers of *P* elements in a genome to GD phenotypes, which found weak or no correlations using Southern blotting or *in situ* hybridization to polytene chromosomes (Todo et al., 1984; Engels, 1984; Boussy et al., 1988; Ronsseray, Lehmann & Anxolabéhère, 1989; Rasmusson et al., 1990; Itoh et al., 1999; Itoh et al., 2004; Itoh et al., 2007; Bonnivard & Higuet, 1999; Itoh & Boussy, 2002). The lack of association between *P* element content and GD phenotypes we observe is unlikely to solely result from noise due to the incomplete inbreeding of the isofemale strains studied here, since a similar lack of strong association was recently reported using the highly inbred DGRP lines (Srivastav & Kelleher, 2017). Assuming that GD assays using single reference strains provide robust insight into the GD phenotypes of these natural strains, our results are at face value consistent with recent arguments that the *P* element may not be the sole determinant of hybrid dysgenesis (Zakharenko & Ignatenko, 2014). However, our results are also consistent with GD phenotypes being determined by the activity of particular *P* elements found at specific locations of the genome (Ronsseray, Lehmann & Anxolabehere, 1991), a possibility that would demand development of new bioinformatics strategies that predict both the structure and location of individual *P* element insertions. Alternatively, the lack of correlation between the *P* element content and GD phenotypes may result from the GD assays having substantial experimental variation across lines, or confounding effects of other variation in biological processes such as repression by the piRNA pathway.

In conclusion, our results show that it is possible to detect clear differences in *P* element insertion profiles among populations using either isofemale strain or pool-seq genomic data. However, interpreting if or how *P* element content relates to GD phenotypes at the strain or population level remains an open challenge.

##  Supplemental Information

10.7717/peerj.3824/supp-1 File S1 Raw data for *P* element abundance and gonadal dysgenesis assays in samples from three worldwide *D. melanogaster* populations Comma separated value (CSV) formatted file with strain name, SRA accession, population, median insert size, average depth of coverage in the unique region of the major chromosome arms, %GD data from A and A* crosses, P-M cytotype status, estimates of the number of predicted *P* elements from TEMP (raw and filtered) and RetroSeq (raw and filtered), normalized coverage, full-length *P* elements (*FP*), and the ratio of truncated to full-length elements ( *KP*∕*FP* ratio) for 50 isofemale strains from three global regions. GD data are taken from Ignatenko et al. (2015) and were standardized to definitions proposed by Engels & Preston (1980) prior to re-analysis here. GD data from Ignatenko et al. (2015) are not available (NA) for seven isofemale strains and six pool-seq samples from Bergman & Haddrill (2015) .

10.7717/peerj.3824/supp-2 File S2 Raw data for locations of *P* elements in strain-specific and pool-seq genome sequences from three worldwide *D. melanogaster* populations Zip archive of browser extensible data (BED) files of predicted *P* element locations in genome sequences from 50 isofemale strains and 6 pool-seq samples from three global regions. Each sample has four BED files corresponding to raw (*raw.bed) and filtered (*nonredundant.bed) output from TEMP and RetroSeq, respectively. BED files for seven isofemale strains from Bergman & Haddrill (2015) are included here that do not have GD data in Ignatenko et al. (2015) but are included in the pool-seq samples, allowing comparisons to be made between isofemale strains and pool-seq samples for the same set of strains.

10.7717/peerj.3824/supp-3 File S3 Raw data for *P* element coverage profiles for strain-specific and pool-seq genome sequences from three worldwide *D. melanogaster* populations Depth of sequencing coverage across the canonical *P* element sequence from 50 isofemale strains and six pool-seq samples from three global regions. Depth of coverage was computed using SAMtools depth and normalized to coverage in the unique regions of the dm6 reference genome.

10.7717/peerj.3824/supp-4 Figure S1 Distribution of *P* element content for isofemale strains within and between three worldwide populations of *D. melanogaster* Distributions for total number of predicted *P* elements are shown as boxplots with black lines representing median values, boxes representing the interquartile range (IQR), whiskers representing the limits of values for strains that lie within 1.5 ×IQR of the upper or lower quartiles, and circles representing strains that lie outside 1.5 ×IQR of the upper or lower quartiles. Numbers of *P* elements predicted by TEMP shown are before (raw) and after (filtered) standard filtering by McClintock and are not weighted by sample frequency. Analogous results for weighted output of TEMP or RetroSeq are shown in Fig. 1.

10.7717/peerj.3824/supp-5 Figure S2 Results of GD tests for isofemale strains from natural populations from N. America, Europe and Africa %GD for cross A (tester strain males versus M-strain Canton-S females, vertical axis) and cross A* (P-strain Harwich males versus tester strain females, horizontal axis) are based on data reported in Ignatenko et al. (2015). Cross A and A* labels in Ignatenko et al. (2015) are inverted relative to those proposed by Engels & Preston (1980) and were converted to standard labels prior to analysis here. Each dot represents an isofemale strain. A shows the P-M status for various sectors of GD phenotypic space defined by A and A* crosses are according to Kidwell, Frydryk & Novy (1983) and Anxolabéhère & Quesneville (1998).

10.7717/peerj.3824/supp-6 Figure S3 Relationship between P element content and GD phenotypes in worldwide sample of *D. melanogaster* isofemale strains from N. America, Europe and Africa %GD data are from Ignatenko et al. (2015) and use the same standardized definitions as in Fig. 2. Numbers of *P* elements predicted by TEMP or RetroSeq shown shown are before (raw) and after (filtered) standard filtering by McClintock. Each dot represents an isofemale strain.
Genomic analysis of *P* elements in natural populations of *Drosophila melanogaster*.

## Additional Information and Declarations

### Data Availability

The following information was supplied regarding data availability: The raw data has been provided as  Supplemental Files.
