# Supplementary figures and images for: Genomic analysis of P elements in natural populations of Drosophila melanogaster
Source: PeerJ. 2017 Sep 15;5:e3824. doi: 10.7717/peerj.3824 (PMC5602686; doi:10.7717/peerj.3824)

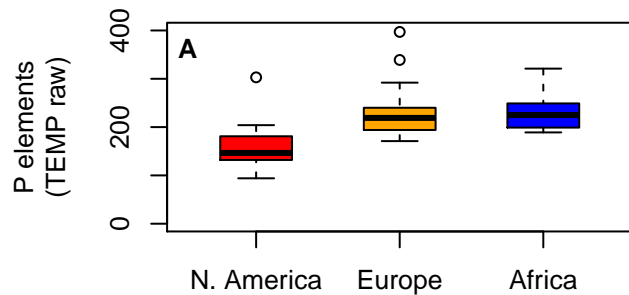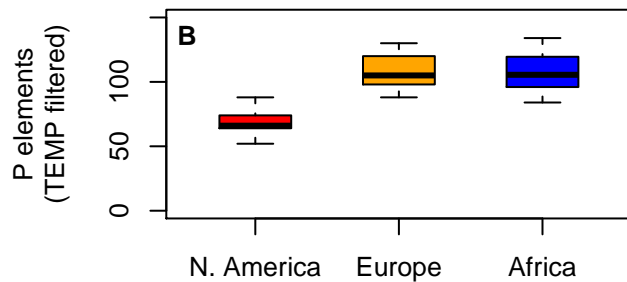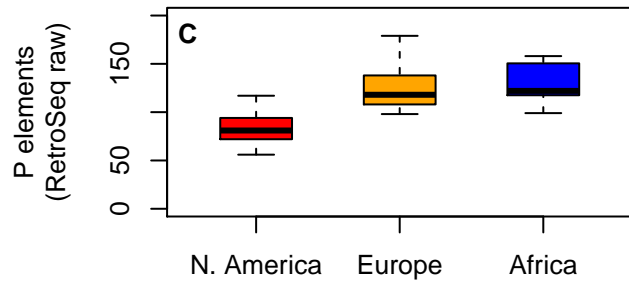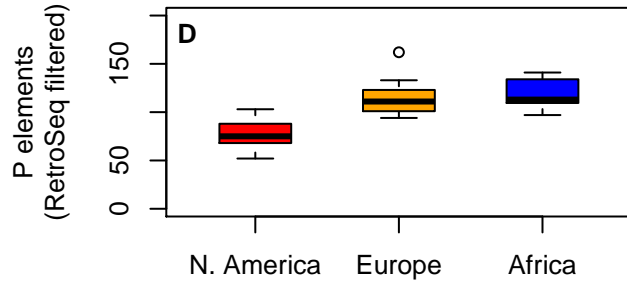

Supplement: Figure S1 — Distributions for total number of predicted P elements are shown as boxplots with black lines representing median values, boxes representing the interquartile range (IQR), whiskers representing the limits of values for strains that lie within 1.5 ×IQR of the upper or lower quartiles, and circles representing strains that lie outside 1.5 ×IQR of the upper or lower quartiles. Numbers of P elements predicted by TEMP shown are before (raw) and after (filtered) standard filtering by McClintock and are not weighted by sample frequency. Analogous results for weighted output of TEMP or RetroSeq are shown in Fig. 1. [file peerj-05-3824-s004.pdf]

• N. America • Europe • Africa

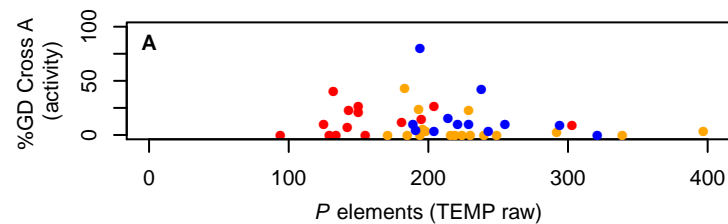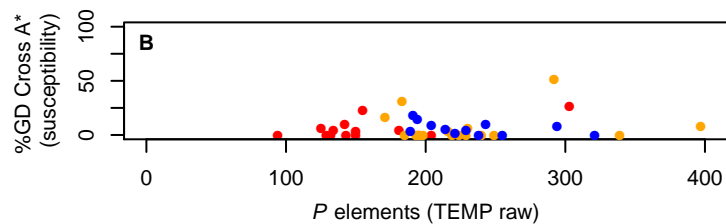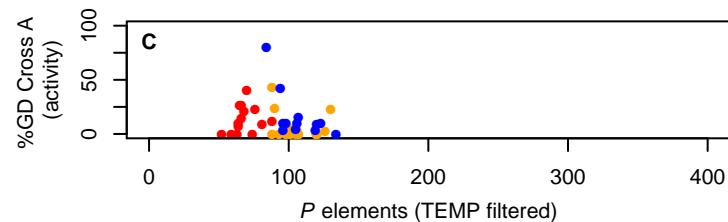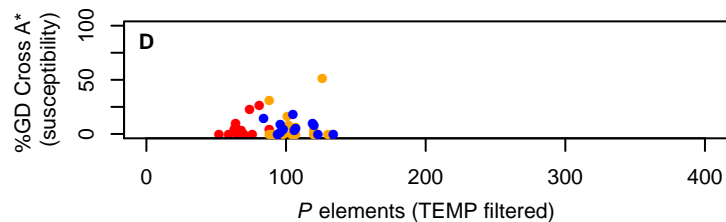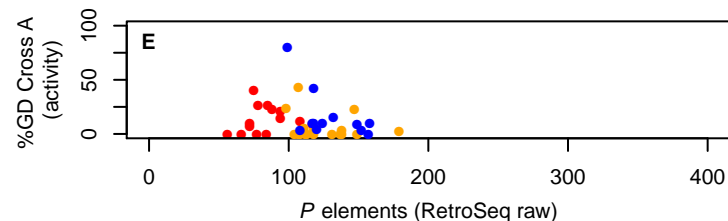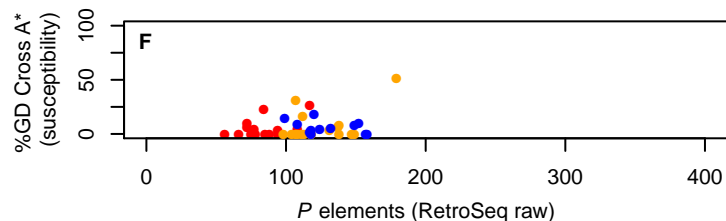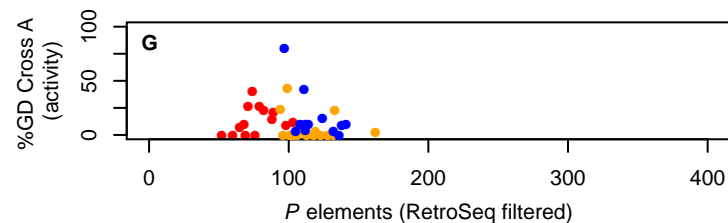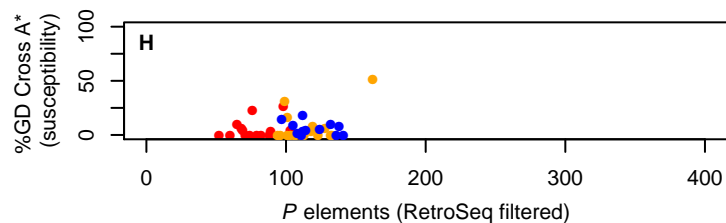

Supplement: Figure S3 — %GD data are from Ignatenko et al. (2015) and use the same standardized definitions as in Fig. 2. Numbers of P elements predicted by TEMP or RetroSeq shown shown are before (raw) and after (filtered) standard filtering by McClintock. Each dot represents an isofemale strain. Genomic analysis of P elements in natural populations of Drosophila melanogaster. [file peerj-05-3824-s006.pdf]
