# Supplementary material for: Genomic analysis of P elements in natural populations of Drosophila melanogaster
Source: PeerJ. 2017 Sep 15;5:e3824. doi: 10.7717/peerj.3824 (PMC5602686; doi:10.7717/peerj.3824)
Supplement: Figure S2 — %GD for cross A (tester strain males versus M-strain Canton-S females, vertical axis) and cross A* (P-strain Harwich males versus tester strain females, horizontal axis) are based on data reported in Ignatenko et al. (2015). Cross A and A* labels in Ignatenko et al. (2015) are inverted relative to those proposed by Engels & Preston (1980) and were converted to standard labels prior to analysis here. Each dot represents an isofemale strain. A shows the P-M status for various sectors of GD phenotypic space defined by A and A* crosses are according to Kidwell, Frydryk & Novy (1983) and Anxolabéhère & Quesneville (1998). [file peerj-05-3824-s005.pdf]

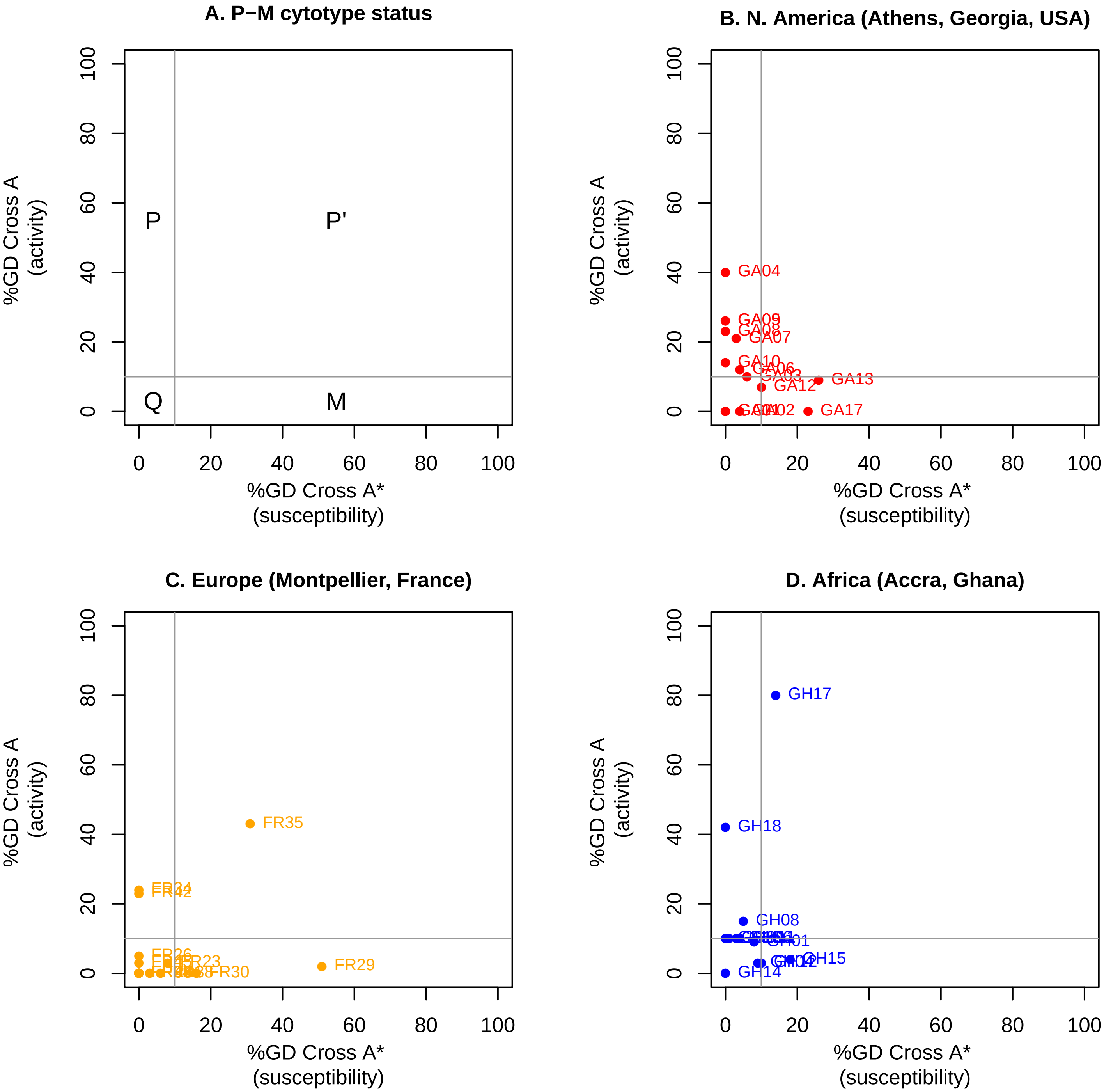
A. P−M cytotype status
B. N. America (Athens, Georgia, USA)
C. Europe (Montpellier, France)
D. Africa (Accra, Ghana)
%GD Cross A
(activity)
%GD Cross A*
(susceptibility)
0
20
40
60
80
100
P
P'
Q
M
GA04
GA07
GA12
GA13
GA17
FR35
FR29
FR30
GH17
GH18
GH08
GH15
GH14
